# Thyroid cancer and COVID-19: experience at one single thyroid disease referral center

**DOI:** 10.1007/s12020-021-02650-z

**Published:** 2021-02-27

**Authors:** Alessandro Prete, Marco Falcone, Valeria Bottici, Carlotta Giani, Giusy Tiseo, Laura Agate, Antonio Matrone, Virginia Cappagli, Laura Valerio, Loredana Lorusso, Elisa Minaldi, Eleonora Molinaro, Rossella Elisei

**Affiliations:** 1 Unit of Endocrinology, Department of Clinical and Experimental Medicine, University of Pisa, 56122 Pisa, Italy; 2 Unit of Infectious Diseases, Department of Clinical and Experimental Medicine, University of Pisa, 56122 Pisa, Italy

**Keywords:** Thyroid cancer, COVID-19, Multikinase inhibitors, QT prolongation, CYP3A4

## Abstract

**Purpose:**

Severe acute respiratory syndrome coronavirus 2 (SARS-Cov-2) is challenging health systems all over the world. Cancer patients have a higher risk of being infected by SARS-Cov-2 and higher coronavirus disease 2019 (COVID-19) severity and mortality. Up to date, there were no data about COVID-19 in patients with thyroid cancer (TCs). The aim of the study was to describe the prevalence of COVID-19 in a well-characterized series of TC patients evaluated for the persistence of the neoplastic disease from March to September 2020; as secondary objective, we looked for the COVID-19 disease severity in a subgroup of multimetastatic TC patients.

**Methods:**

We evaluated 1464 patients affected by persistent TC: 67 patients who were taking multikinase inhibitors (MKIs) and 1397 under active surveillance for a persistent but stable disease. During the clinical evaluation, all patients were specifically investigated about a positive history of Sars-Cov-2 infection.

**Results:**

SARS-Cov-2 infection was identified in 4/1464 (0.3%) cases of patients affected by TC. We identified three cases among patients under active surveillance (0.2%), and one case among patients treated with MKI systemic therapy (1/67, 1.5%). This patient was taking vandetanib for metastatic medullary thyroid cancer (MTC), when he came to our attention referring severe fatigue, dyspnea for light physical activities. He presented a mild COVID-19 and he received exclusively supportive care. After a multidisciplinary consultation, we decided against the discontinuation of vandetanib. After 2 months from the infection, he did not present any signs of active infection, and the MTC metastatic disease was stable.

**Conclusions:**

We showed that COVID-19 is not more frequent in TC patients than in general population, although a relatively higher prevalence in the group of TC patients treated with MKIs. A single patient with advanced TC and SARS-Cov-2 infection during MKIs treatment had a mild COVID-19 and did not require the discontinuation of MKI therapy. In cases of more severe COVID-19, an accurate evaluation from a multidisciplinary team would consider risks and benefits in taking the decision to continue or stop MKI treatment.

## Introduction

On March 11, 2020, severe acute respiratory syndrome coronavirus 2 (SARS-Cov-2) infection outbreak was declared as pandemic by the World Health Organization. As of November 25, 2020, more than 59 million of infected patients have been registered globally, and more than 1,390,000 patients passed away after virus infection disease, now known as SARS-Cov-2. According to the most recent data, coronavirus disease 2019 (COVID-19) presents a mortality rate of 1.38% [1], even higher in old patients and in patients with multiple comorbidities [2].

Cancer patients are vulnerable patients, mostly due to their multiple comorbidities. Fowler et al. [3] have recently showed that at least one comorbidity was present in up to two-thirds of neoplastic patients, and roughly half of the patients had multiple diseases. On one hand, hypertension, chronic obstructive pulmonary disease, and diabetes were the most frequent diseases in cancer patients [3], and, in the other hand, all of them were related to a higher risk of mortality in patients affected by COVID-19 [2, 4–6].

According to their “frail” status, neoplastic patients demonstrated to have higher risk of being infected by SARS-Cov-2 [7]. Moreover, they deteriorate faster into severe illness and present higher severity and mortality of COVID-19, than those without cancer [8]. Since, neoplastic patients with active/progressive diseases presented higher risk of mortality [9], the impact of anticancer therapies on COVID-19 mortality was questioned by many clinicians.

After 6 months from the begin of COVID-19 pandemic in Italy, we investigated the impact of COVID-19 in patients with thyroid cancer (TC) with particular regard to those with an advanced and multimetastatic disease who were taking multikinase inhibitors (MKIs) as systemic antineoplastic therapy. Main aim of this study was to describe the prevalence of COVID-19 in our series of biochemical and structural persistent TC patients evaluated at the endocrine-oncology unit of our University Hospital from March to September 2020; additionally, we described the COVID-19 disease severity in a subgroup of multimetastatic TC patients who would be theoretically at higher risk to develop medical complication from COVID-19. In particular, we concentrated our attention on those metastatic patients treated with MKIs to analyze the possible interactions between MKIs and anti-COVID-19 drugs.

## Results

### Patients' evaluation

Thank to this survey, we discovered 4/1464 (0.3%) cases of patients affected by TC who had SARS-Cov-2 infection. We identified three cases among patients under active surveillance, and one case among patients treated with MKI systemic therapy (1/67, 1.5%). The three patients with TC under active surveillance developed a mild COVID-19 and none of them required hospitalization. No worsening of biochemical and/or structural neoplastic disease was observed after COVID-19.

Epidemiological, clinical, and pathological features of patients with metastatic TC who were taking MKIs are summarized in Table 1. The median age was 62.5 years and 41 patients were male (61.2%). About half patients were affected by medullary thyroid cancer (MTC), while 18.1%, 16.7%, and 9.7% patients presented papillary (PTC), follicular (FTC), and poorly differentiated (PDTC) TC, respectively. Lung metastases were present in 71.6% patients. In all, 65.7% patients had cardiovascular comorbidities, while only 7.5% and 6.2% had pulmonary comorbidities and diabetes, respectively. A small part of them (13.4%) presented ECOG status more than 1.

**Table 1:** Epidemiological and clinical-pathologic features of 67 metastatic thyroid cancer patients

	67 patients
Age at time of last evaluation (years) (median) [IQR, intervals]	62.5 [52–71.75, 28–91]
Sex	26 female: 41 male
Histotypes—no (%)	
MTC	35 (48.6%)
PTC	13 (18.1%)
FTC	12 (16.7%)
PDTC	7 (9.7%)
Metastatic sites—no (%)	
Lymph node	56 (83.6%)
Lung	48 (71.6%)
Liver	30 (45.5%)
Bone	27 (40.3%)
Central nervous system	9 (13.6%)
Cardiovascular comorbidities—no (%)	
Total	44 (65.7%)
Major adverse cardiovascular events	12 (17.9%)
Hypercholesterolemia	8 (11.9%)
Arterial hypertension	38 (56.7%)
Diabetes—no (%)	5 (7.5%)
Asthma/COPD—no (%)	4 (6.2%)
ECOG status at last evaluation—no (%)	
Grade 0	35 (52.2%)
Grade 1	23 (34.3%)
Grade 2	9 (13.4%)
Surgery—no (%)	
Thyroidectomy	26 (39.4%)
Thyroidectomy + lymph node dissection	40 (60.6%)
Radiotherapy—no (%)	23 (34.3%)
Radioiodine therapy—no (%)	32 (48.5%)
Radioiodine cumulative dose (mCi)	
Median	278
IQR	127.75–561.25
Intervals	30–849.5
Actual MKI—no (%)	
Vandetanib	25 (34.7%)
Lenvatinib	29 (40.3%)
Cabozantinib	4 (5.6%)
Selpercatinib	8 (11.1%)
Sunitinib	1 (1.4%)
Number of MKIs for each patient—no (%)	
1	51 (76.1%)
2	8 (11.9%)
3	7 (10.4%)
4	1 (1.5%)
Total duration of MKIs treatment (months) (median) [IQR, intervals]	46 [23–108, 3–156]
Duration of actual MKI (months) (median) [IQR, intervals]	32 [12–96, 2–156]

*IQR* interquartile range, *MTC* medullary thyroid cancer, *PTC* papillary thyroid cancer, *FTC* follicular thyroid cancer, *PDTC* poorly differentiated thyroid cancer, *COPD* chronic obstructive pulmonary disease, *MKIs* Multikinase inhibitors

Most of the patients were taking lenvatinib (40.3%) or vandetanib (34.7%), while only a few patients were taking cabozantinib (5.6%), selpercatinib (11.1%), or sunitinib (1.4%). At October 2020, the median duration of MKIs treatment was 32 months.

### The COVID-19 patient taking MKIs

On May 7, 2020, after 18 months of vandetanib treatment (200 mg/day), a 64-year-old man affected by an MTC with multiple metastases in cervical and mediastinal lymph nodes, lungs, liver, and bone came to our attention reporting severe fatigue, dyspnea for light physical activities, and a remarkable loss of weight (−6 Kg in 2 months). Otherwise, he denied fever, cough, and further symptoms. At physical examination, he presented only tachypnea. He also reported that, 2 months before, his mother had got SARS-Cov-2 infection and she passed away, after 1 month of hospitalization.

**Fig. 1:**
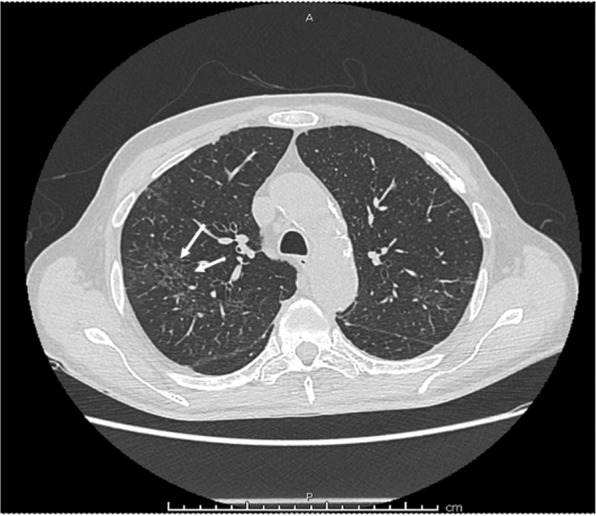
Lung CT scan of the patient with COVID-19 and multimetastatic medullary thyroid cancer taking vandetanib: the interstitial thickening of the right lung, indicated by the white arrows, documented the mild pneumonia that the patient developed during the COVID-19

Based on his clinical presentation and history, after excluding a worsening of the neoplastic disease, suspecting COVID-19 he was sent to the emergency department. On arrival, he presented respiratory alkalosis (pH 7.51, pCO2 26 mmHg, pO2 104 mmHg, and HCO3^−^ 20.7 mmol/L) and optimal O_2_ saturation (96.9%). As supposed, nasopharyngeal swab was positive for SARS-Cov-2 infection. Computer tomography (CT) scan documented the appearance of interstitial thickening of the right lung, in addition to the well-known multiple lung metastasis (Fig. 1). Because the COVID-19 was not severe and the lung involvement was mild, after a multidisciplinary meeting, it was decided to avoid any antiviral or immunomodulatory treatment for COVID-19, but only supportive treatment so that he could continue vandetanib treatment. After a significant improvement of his medical conditions and two negative nasopharyngeal swabs, he was discharged. Two months after the diagnosis, he did not present any sign of relapse, a new nasopharyngeal swab was negative, and the MTC metastatic disease was stable.

## Discussion

SARS-Cov-2 infection had a terrifying impact on all the world. More than 59 million of people got SARS-Cov-2 infections with more than 1,390,000 deaths. In face of the COVID-19 pandemic, researchers are urgently called to provide new insights useful to guide the clinical behavior.

Since the first reports, cancer patients were discovered at higher risk of infection and mortality of COVID-19. Yu et al. [7] found a SARS-Cov-2 infection rate of 0.79% in cancer patients that was higher than the cumulative incidence of all diagnosed COVID-19 over the same time period in Wuhan (0.37%). As of September 13, 2020, 287,753 cases (0.47%) of SARS-Cov-2 infections were reported in Italy, according to institutional data. In our cohort of 1464 advanced TC patients, we reported only four cases of COVID-19 (0.27%). Our data did not show a higher prevalence of SARS-Cov-2 infection in patients with TC compared to Italian general population. However, when we focused our attention on the 67 TC metastatic patients taking MKIs the prevalence appeared higher than the previous one (1/67, 1.5%).

During the observational period, 4 of our 67 advanced TC patients died for disease progression but no one died for COVID-19. About COVID-19 mortality in cancer patients, in a cohort of 44,672 of patients, the history of neoplastic disease was described as a significant higher risk of mortality (RR **=** 2.93, *p* value = 0.006) [10]. However, in neoplastic patients, the COVID-19-related mortality seemed to be principally driven by older age, male gender and presence of comorbidities [11]. In our series, the median age was 62.5 years, the majority of patients were males (61.2%), and many of them had comorbidities (65.7% cardiovascular and 7.5% pulmonary comorbidities, and 6.2% diabetes). However, no COVID-19-related deaths were observed; this might be due to the efficacy of social health measures (e.g., lockdown) performed in Italy to protect frail populations.

Different kinds of cancer as well as different metastatic sites seemed to produce a different impact on the severity of COVID-19. Patients with hematologic cancers (such as leukemia, lymphoma, and myeloma), with lung cancer, and with lung metastasis had higher risk of death, intensive care unit (ICU) admission, severe symptoms, and use of invasive mechanical ventilation [12]. Moreover, among neoplastic patients, those with active/progressive neoplastic disease had higher risk of 30-day all-cause mortality, as well as patients with ECOG status superior than 2 [9]. On this regard, it is worth to note that disease progression of metastatic TC is usually very slow and can be stopped by the MKIs treatment thus likely reducing the susceptibility to a worse COVID-19; likewise, most of TC patients have ECOG 0-1, not only those who are under active surveillance but also the majority of those under systemic therapy [13].

Since patients with active/progressive disease present higher mortality, the impact of anticancer therapies on COVID-19 mortality came out to the attention and it was addressed by many reports and questioned by many clinicians. For this reason, a multidisciplinary meeting was performed to decide if to continue or not the vandetanib therapy in our COVID-19 affected patient. In a recent article by Lee et al. [11], 22% of cancer patients affected by COVID-19 interrupted their antineoplastic treatments due to COVID-19. However, patients who received anticancer therapy during the last 4 weeks before SARS-Cov-2 infection did not show a higher risk of mortality [11]. Likewise, Kuderer et al. [9] analyzed data from the COVID-19 and Cancer Consortium database, which involves patients with active or previous malignancy and COVID-19 from USA, Canada, and Spain. In this cohort of 928 patients, antineoplastic systemic therapy administered until the evidence of SARS-Cov-2 infection did not induce any further risk of mortality. Other reports showed an higher risk of death in patients recently treated with immunotherapy, and of severe/critical disease and ICU admission in patients recently submitted to surgery or treated with immunotherapy [12]. An ESMO Interdisciplinary Expert Consensus suggested to do not treat patients with immunotherapy in case of SARS-Cov-2 infection and to reconsider this treatment after a complete infection resolution [14]. On the other hand, targeted therapies seem to do not increase risk of disease mortality and severity [11, 12]. Based upon these evidences, many oncologists suggested to treat patients with oral systemic therapies instead of immunotherapy or chemotherapy in order to restrict the access of cancer patients to the hospital [15, 16]. According to these reported data and considering the mild severity of the COVID-19 in our patient, we decided to continue the MKI at least until the evidence of a worsening of the COVID-19 that fortunately did not happen.

In order to investigate if it could be reasonably safe to continue MKI in our patient during COVID-19 at same or potentially lower dosage, especially if the COVID-19 would become worse, we evaluated the presence of adverse effects (AEs) in common between drugs that had been used against COVID-19 and vandetanib. By performing this evaluation, we took the opportunity to expand this analysis to the other MKIs used in the clinical practice for the treatment of the different types of TC: cabozantinib, lenvatinib, selpercatinib, and sorafenib [17–24] (Table 2). As expected, symptoms concerning gastrointestinal system (e.g., nausea, vomiting, and diarrhea) were the most diffuse AEs in common between anti-COVID-19 therapies and MKIs and should be monitored. Cutaneous diseases such as skin rash or photosensitivity reaction were also common. Therefore, we investigated the presence of drug interactions between anti-COVID-19 drugs and MKIs (Table 3), using an online database [25]. We found that the main drug interactions are concerning QT prolongation and CYP3A4 inhibition.

**Table 2:** Mechanisms of action, labeled use, metabolic interactions, adverse reactions, and adverse effects in common between anti-COVID-19 drug and multikinase inhibitors (MKIs)

Drug	Mechanism of action	Labeled use	Metabolic interactions	Main adverse effects	Adverse effects in common with MKIs
Chloroquine/hydroxychloroquine	Targeting endosomal acidification, and inhibition binding with ACE2 receptor	Malaria, extra intestinal amebiasis, lupus erythematosus, rheumatoid arthritis	Metabolized by CYP2C8, CYP3A4, CYP2D6	GI: metallic taste, nausea, vomiting, abdominal cramping, and diarrhea CV: cardiomyopathy and QTc prolongation Cutaneous: photosensitivity and lichenoid reactions Miscellaneous: neuropathy, headache, myopathy, and retinopathy	Nausea, vomiting, abdominal cramping, diarrhea, QTc prolongation, and photosensitivity
Azithromycin	Enhancement of the anti-SARS-Cov-2 activity of hydroxychloroquine	Chancroid, COPD acute exacerbation, mycobacterium avium complex infection, acute otitis media, community-acquired pneumonia, uncomplicated skin infection, streptococcal pharyngitis, and urethritis/cervicitis	Moderate inhibitor of CYP3A4	GI: nausea, vomiting, diarrhea, and increase of AST and ALT Miscellaneous: skin rash, increase of BUN and creatinine, and QT prolongation	Nausea, vomiting diarrhea, QT prolongation, and skin rash
Remdesivir	Inhibiting RNA-dependent RNA polymerase	Indicated for the treatment for COVID-19 patients with pneumonia needing oxygen supplementation but not for patients needing high-flow oxygen, noninvasive or invasive mechanical ventilation or extracorporeal membrane oxygenation	–	GI: nausea, vomiting, gastroparesis, rectal bleeding, and AST elevation Miscellaneous: hypomagnesemia, skin rash, and hypotension	Nausea, vomiting, gastrointestinal bleeding, and skin rash
Favipiravir		Not indications so far	–	GI: decreased appetite, nausea, vomiting, diarrhea, and increase of AST and ALT	Decreased appetite, nausea, vomiting, and diarrhea
Ribavirin	Inhibition of viral RNA synthesis	HCV infection	Metabolized by CYP enzyme pathways	GI: xerostomia, anorexia, nausea, vomiting, abdominal pain, and diarrhea Electrolytic balance: hypocalcemia and hypomagnesemia Cutaneous: dermatitis and skin rash Miscellaneous: flu-like syndrome, anemia, hemolytic anemia, and hypothyroidism	Xerostomia, anorexia, nausea, vomiting, abdominal pain, diarrhea, dermatitis, skin rash, and hypothyroidism
Lopinavir/ritonavir	Inhibiting protease activity	HIV infection	strong inhibitor of CYP3A4, moderate inhibitor of p-glycoprotein (P-gp), organic anion transporter (OATP1B1), OATP1B3, inducer of UDP-glucuronosyltransferase	GI: nausea, vomiting, diarrhea, and increase of AST, ALT, and amylase	Nausea, vomiting, and diarrhea
Nitazoxanide	Inhibition of viral replication	Cryptosporidium parvum/giardia lamblia diarrhea	–	GI: nausea, gastroesophageal reflux disease, abdominal pain, and diarrhea Miscellaneous: headache, discoloration of eyes and urine, dizziness, skin rash, and urticaria	Nausea, abdominal pain, diarrhea, and skin rash
Nelfinavir		HIV infection	Metabolized by CYP3A4 and CYP2C19	GI: diarrhea and nausea Miscellaneous: skin rash	Diarrhea, nausea, and skin rash
Tocilizumab	Immunomodulatory effects via inhibition interleukin-6	Cytokine release syndrome, giant cell arteritis, polyarticular juvenile idiopathic arthritis, rheumatoid arthritis, and systemic juvenile idiopathic arthritis	Increase of CYP1A2, CYP2B6, CYP2C9, CYP2C19, CYP2D6, and CYP3A4 activity	GI: stomatitis, diarrhea, gastrointestinal perforation, and increase of AST and ALT Miscellaneous: infections, hypertension, headache, skin reactions, hypertension, and hypothyroidism	Stomatitis, diarrhea, gastrointestinal perforation, skin reactions, hypertension, and hypothyroidism
Interferon-Alpha	Immunomodulatory effects	ADIS-related Kaposi sarcoma, chronic hepatitis B, condylomata acuminata, follicular lymphoma, hairy cell leukemia, and melanoma	–	GI: xerostomia, anorexia, nausea, vomiting, abdominal pain, diarrhea, increase of AST and ALT, and autoimmune hepatitis Miscellaneous: flu-like symptoms, skin rash, leukopenia, and lymphopenia	Xerostomia, anorexia, nausea, vomiting, abdominal pain, diarrhea, flu-like symptoms, and skin rash
Baricitinib	Immunomodulatory effects via JAK inhibition	Rheumatoid arthritis FDA approved emergency use for COVID-19 (https://www.fda.gov/news-events/press-announcements/coronavirus-covid-19-update-fda-authorizes-drug-combination-treatment-covid-19)	Substrate of P-gp, breast cancer resistance protein OAT, and multidrug and toxin extrusion protein transporters	Miscellaneous: infections, thrombosis, neutropenia, platelet elevations, elevation of ALT, AST and creatinine, and skin rash	Thrombosis and skin rash

*ACE2* angiotensin-converting enzyme 2, *CYP* cytochrome P450, *GI* gastrointestinal, *CV* cardiovascular, *AST* aspartate aminotransferase, *ALT* alanine aminotransferase, *OATP* organic anion transporting polypeptide, *JAK2* Janus kinase 2

**Table 3:**
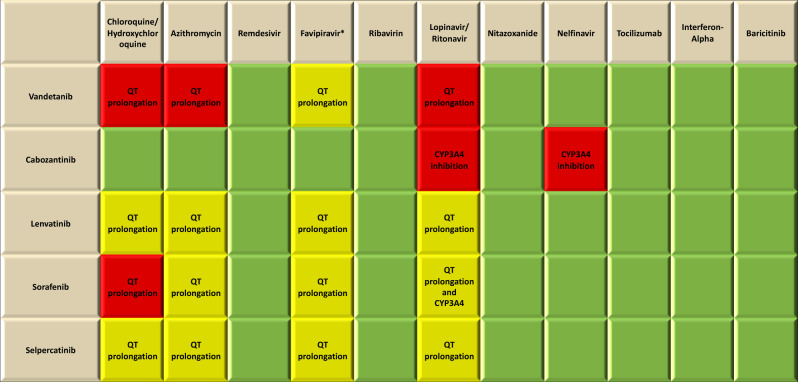
Main drug interactions between each anti-COVID-19 drug and each multikinase inhibitors (MKIs)

^a^Favipiravir was not present in the online database and its QT-prolonging effect was documented by ref. [19]

The interactions inducing QT prolongation between vandetanib and chloroquine/hydroxychloroquine, azithromycin, or lopinavir/ritonavir should induce the clinicians to stop vandetanib treatment. Likewise, sorafenib should be stopped in case of chloroquine/hydroxychloroquine therapy. As general rule, caution about QT prolongation must be had in case of treatment with all MKIs whenever an anti-COVID-19 treatment must be started by referring to a recent consensus [26]. If it is not possible to avoid QTc-prolonging agent (e.g., MKIs), baseline EKG should be performed, any structural heart disease excluded, and any causes of QT prolongation such as hypokalemia, hypomagnesemia, fever, and an inflammatory state should be avoided. During the treatment, periodic EKG should be performed and, in case of prolonged QT (>500 ms), cardiologic consult should be performed and the QTc-prolonging agent (e.g., MKI) should be interrupted [26] having in mind that the neoplastic disease could explode after the antineoplastic drug suspension [27].

The other possible drug interaction is concerning CYP3A4 inhibition that could decrease the MKI metabolism and increase the risk of AEs. Cabozantinib therapy should be stopped in case of treatment with lopinavir/ritonavir or with nelfinavir, in order to avoid the increase of AEs risk [28]. Similarly, caution should be used if lopinavir/ritonavir is employed in patient treated with sorafenib.

## Materials and methods

Between March and September 2020, a total of 1464 patients affected by TC were evaluated at the Endocrine Unit of the University Hospital of Pisa. According to the most recent guidelines [29, 30], we performed a fully comprehensive neoplastic revaluation. Among them, there were 67 patients who were taking MKIs and 1397 who were affected by a persistent biochemical or structural TC [29, 30] with stable or slowly progressive disease and who were under active surveillance without taking any systemic treatment. In this specific period of time, cured patients were followed with telephone calls and were not included in this study.

During the clinical evaluation, all patients were specifically investigated about a positive history of SARS-Cov-2 infection (history of positive nasopharyngeal swab or presence of antibodies against SARS-Cov-2 epitopes). Moreover, on October 2020 we reassessed all the 67 patients taking TKIs by phone questionnaire.

## Conclusions

This study shows that COVID-19 is not more frequent in TC patients than in general population, although a relatively higher prevalence was found in the restricted group of TC patients treated with MKI. The single case of advanced TC with COVID-19 during MKIs treatment had a mild COVID-19 and did not require the suspension of the systemic antineoplastic therapy. A more severe COVID-19 disease might require a MKI-reduced dosage or suspension; however, the decision is certainly difficult, because the risk of a rapid progression of the malignancy would be very high, and require an accurate evaluation from a multidisciplinary team who would consider all risks and benefits in taking the decision. According to our clinical experience with MKIs, if possible, the reduction of the daily dosage should be preferred respect to the withdrawal if anti-COVID-19 drugs are needed.
